# Transarterial Embolization of Type 2 Endoleak Post Thoracic Endovascular Aortic Repair (TEVAR) Using a Triaxial System With a 1.3-F Microcatheter: A Case Report

**DOI:** 10.7759/cureus.51694

**Published:** 2024-01-05

**Authors:** Yasuyuki Onishi, Hironori Shimizu, Masahide Kawatou, Kenji Minatoya, Yuji Nakamoto

**Affiliations:** 1 Diagnostic Imaging and Nuclear Medicine, Kyoto University, Kyoto, JPN; 2 Cardiovascular Surgery, Kyoto University, Kyoto, JPN

**Keywords:** distal access catheter, embolization, triaxial system, thoracic endovascular aortic repair, type 2 endoleak

## Abstract

Although transarterial embolization is recognized as a treatment for type 2 endoleaks, it can occasionally be challenging. We report the case of an 86-year-old man who presented with an enlarging thoracoabdominal aortic aneurysm following thoracic endovascular aortic repair. Using a triaxial system with a 1.3-F microcatheter, transarterial embolization of a type 2 endoleak was successfully performed through a long and tortuous arterial route comprising the thoracodorsal and ninth intercostal arteries. The postoperative clinical course was uneventful, and computed tomography obtained six days later showed no endoleak in the thoracoabdominal aortic aneurysm. This case suggests the usefulness of a triaxial system with a 1.3-F microcatheter for transarterial embolization of type 2 endoleaks.

## Introduction

Type 2 endoleaks (T2ELs) occur in 3.3% of patients following thoracic endovascular aortic repair (TEVAR) [1]. They result from the retrograde flow into the aneurysm sac from the left subclavian, intercostal, bronchial, and visceral arteries [2]. If the aneurysm sac size increases in patients with T2EL from the left subclavian artery, the proximal subclavian artery should be embolized [3]. In contrast, the treatment of T2EL from other vessels is challenging [3,4], and a few case reports have described transarterial embolization for T2EL after TEVAR [5-7]. Here, we report the case of a T2EL post TEVAR due to retrograde flow from the intercostal artery, in which transarterial embolization was successfully performed using a triaxial system with a 1.3-F microcatheter.

## Case presentation

**Figure 1:**
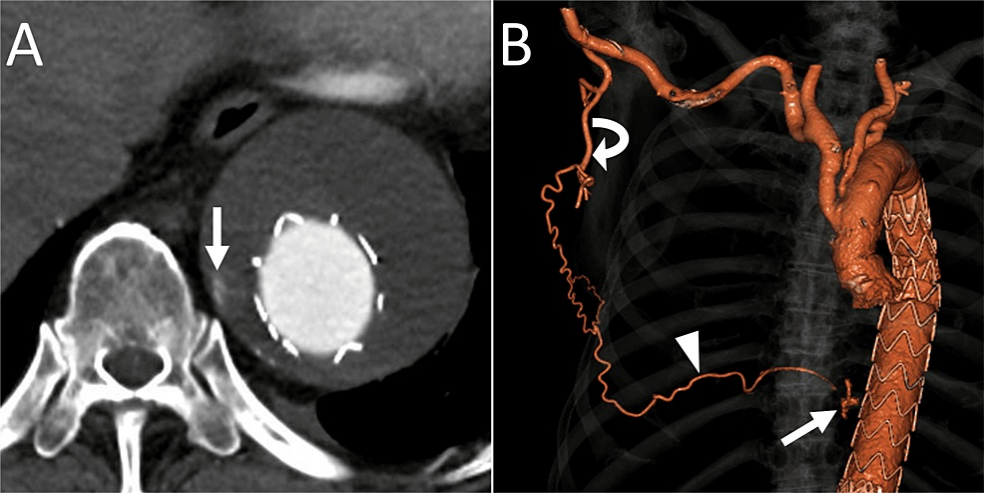
Contrast-enhanced CT before transarterial embolization for a type 2 endoleak. (A) Axial arterial phase contrast-enhanced CT image of the lower thorax showing an endoleak (arrow). (B) Anterior view of three-dimensional CT showing a type 2 endoleak (arrow) through the thoracodorsal artery (curved arrow) and the ninth intercostal artery (arrowhead). CT: computed tomography

The patient was an 86-year-old man with a history of multiple aortic surgeries. Twelve years prior, he underwent ascending aorta and arch replacement for a thoracic aortic aneurysm, and seven years prior, he underwent TEVAR for a thoracoabdominal aortic aneurysm. A recent computed tomography (CT) showed an 8-cm-diameter thoracoabdominal aortic aneurysm, which was 1 cm larger than the CT obtained one year prior. CT also revealed a T2EL through the right ninth intercostal artery (Figure 1). No other endoleaks were observed.

**Figure 2:**
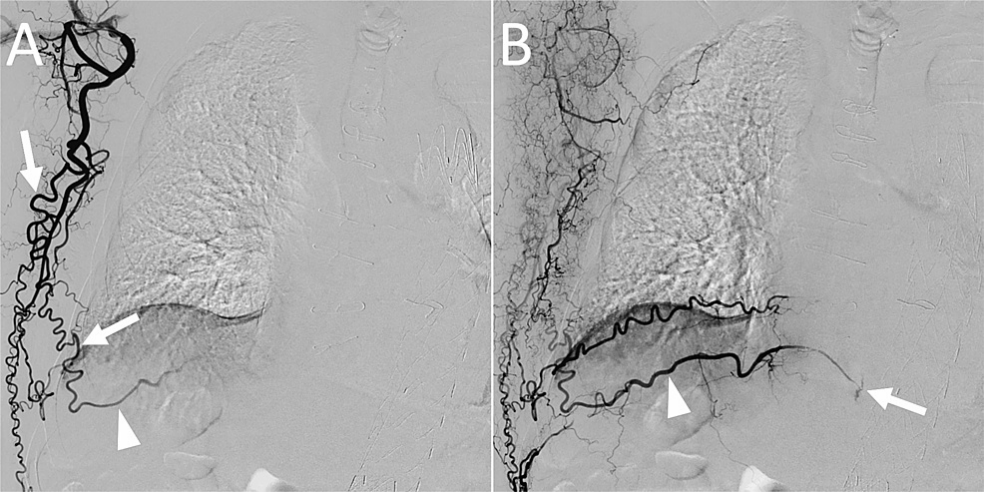
Angiography of the thoracodorsal artery before transarterial embolization for a type 2 endoleak. (A) Early arterial phase image showing a long and tortuous branch of the thoracodorsal artery (arrows) communicating with the ninth intercostal artery (arrowhead). (B) Late arterial phase image showing a type 2 endoleak (arrow) from the ninth intercostal artery (arrowhead).

We decided to treat T2EL because the aortic aneurysm was large and it showed rapid growth. The right ninth intercostal artery communicated with the thoracodorsal artery, and transarterial embolization was planned for the endoleak. As the arterial route to the endoleak cavity was long and tortuous, we used a triaxial system comprising large and small microcatheters. Embolization was performed under local anesthesia and moderate sedation. The right brachial artery was punctured, and a 4-F guiding sheath with an effective length of 50 cm (Parent Plus 30, Medikit, Tokyo, Japan) was placed. The right thoracodorsal artery was selected using a 4-F cobra catheter. Angiography of the right thoracodorsal artery revealed a long and tortuous branch communicating with the right ninth intercostal artery and a T2EL through the intercostal artery (Figure 2).

**Figure 3:**
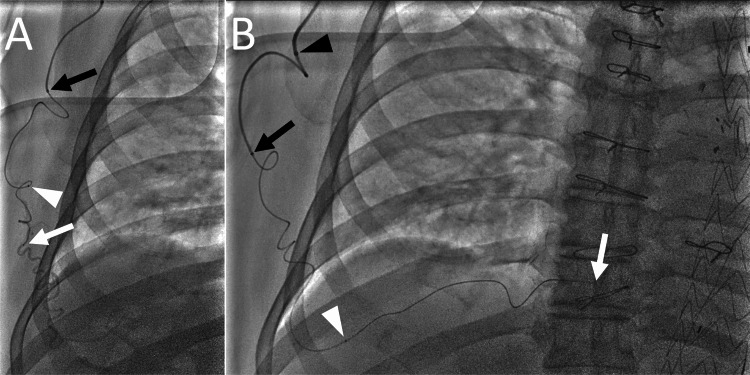
Fluoroscopic image during transarterial embolization for a type 2 endoleak. (A) Angiography of the 1.6-F microcatheter showing the tortuosity of the thoracodorsal artery branch. Note the tip positions of the 1.6-F microcatheter (white arrow), 2.6-F microcatheter (white arrowhead), and 4-F distal access catheter (black arrow). At that point, a 0.014-inch guidewire could not be advanced further because of the high friction between the guidewire and the 1.6-F microcatheter. (B) After changing the 1.6-F microcatheter to a 1.3-F microcatheter, the catheters were successfully advanced using a 0.010-inch guidewire. Note the tip positions of the 1.3-F microcatheter (white arrow), 2.6-F microcatheter (white arrowhead), 4-F distal access catheter (black arrow), and 4-F guiding sheath (black arrowhead).

The cobra catheter was exchanged with a 4-F distal access catheter (4F Cerulean G, Medikit, Tokyo, Japan), and a large microcatheter with a distal diameter of 2.6-F and an effective length of 125 cm (Carnelian HF-S, Tokai Medical Products, Aichi, Japan) was advanced from the 4-F catheter. Coaxially, through the large microcatheter, a small microcatheter with a distal diameter of 1.6-F, a proximal diameter of 1.8-F, and an effective length of 155 cm (Carnelian Marvel S, Tokai Medical Products, Aichi, Japan) was advanced using a 0.014-inch guidewire to reach the endoleak cavity. During the procedure, however, the guidewire could not be advanced further because of the high friction between the guidewire and the microcatheter due to the tortuosity of the vessel (Figure 3A). Therefore, we decided to use a smaller microcatheter and guidewire. The small microcatheter was exchanged for a different small microcatheter with a distal diameter of 1.3-F, a proximal diameter of 1.8-F, and an effective length of 155 cm (Carnelian Marvel S 1.3, Tokai Medical Products, Aichi, Japan). The inner diameter of the catheter was 0.011 inches. A 0.010-inch guidewire was advanced from the 1.3-F microcatheter, and the wire easily passed through the tortuous communicating artery. The 1.3-F and 2.6-F microcatheters and the 4-F distal access catheter were advanced deeply (Figure 3B).

**Figure 4:**
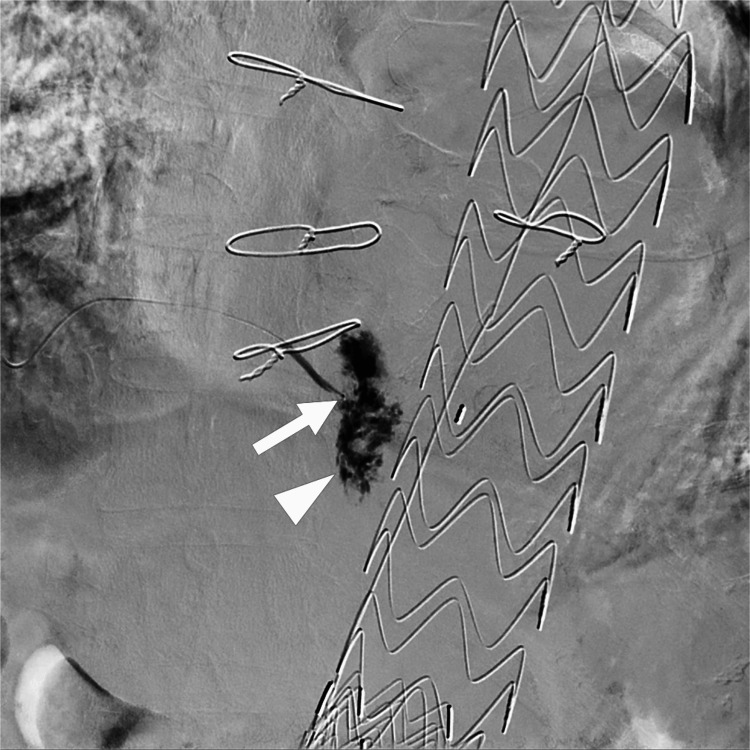
Angiography from the 1.3-F microcatheter (arrow) shows the endoleak cavity (arrowhead). No inflow or outflow vessels are observed.

The 1.3-F microcatheter reached the endoleak cavity, and angiography revealed no visualization of the inflow and outflow vessels (Figure 4).

**Figure 5:**
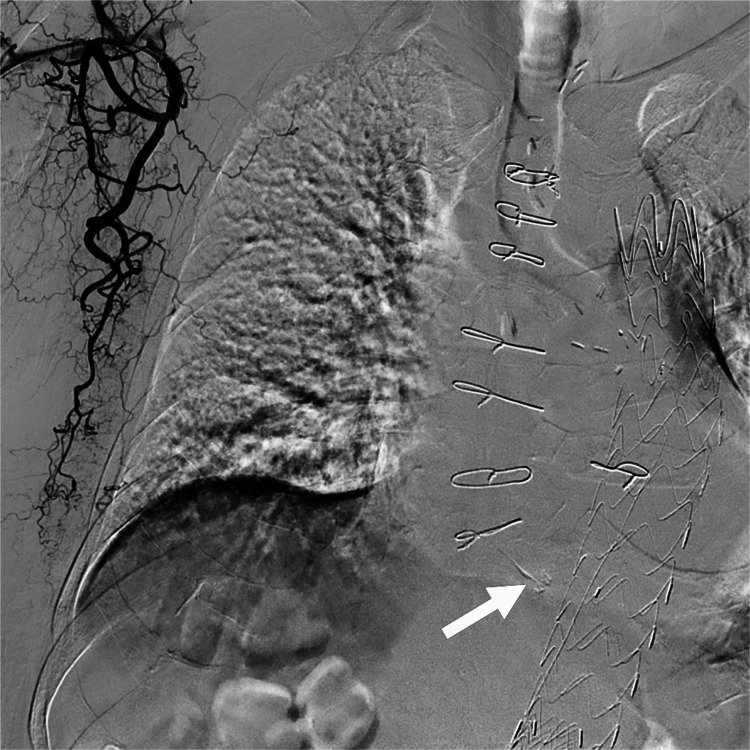
Angiography of the thoracodorsal artery after transarterial embolization shows no visualization of a type 2 endoleak. NBCA cast (arrow) is observed in the endoleak cavity and the ninth intercostal artery. NBCA: n-butyl cyanoacrylate

The endoleak cavity and the ninth intercostal artery were embolized using an n-butyl cyanoacrylate (NBCA) and lipiodol mixture (1:5, 0.5 mL). Angiography of the right subscapular artery revealed endoleak occlusion (Figure 5).

The patient experienced an uneventful postoperative clinical course. CT obtained six days later showed no endoleak in the thoracoabdominal aortic aneurysm.

## Discussion

Previous studies have reported the usefulness of a triaxial system using a 2.7-F large microcatheter and a 1.6-F or 1.9-F small microcatheter coaxially for the embolization of a T2EL after endovascular abdominal aortic aneurysm repair [8-10]. The large microcatheter provides stability to the small microcatheter, allowing deeper advancement of the small microcatheter [9]. After the advancement of the small microcatheter, it acts as a thick guidewire, enabling further advancement of the large microcatheter [11]. Subsequently, the deeply advanced large microcatheter provided further stability to the small microcatheter. In this case, the conventional triaxial system with a 1.6-F microcatheter did not work well because of the tortuosity of the artery. Thus, a 1.3-F small microcatheter was advanced from the large microcatheter over a 0.010-inch guidewire, and the aneurysm sac was reached. We consider that the flexibility and trackability of a 1.3-F small microcatheter and 0.010-inch guidewire are higher than those achieved by a 1.6-F small microcatheter and 0.014-inch guidewire and that a triaxial system with a 1.3-F small microcatheter is a useful tool for T2EL embolization.

A distal access catheter has a highly flexible distal tip and a supportive proximal shaft, providing high trackability and stability for the microcatheter [12,13]. In this case, the distal access catheter worked synergistically with a triaxial system using a 1.3-F microcatheter to overcome the tortuosity of the arterial route to the endoleak cavity. We recommend this combination for treating T2EL when the arterial route is long and tortuous.

A triaxial system with a 1.3-F small microcatheter has several disadvantages. First, because of its small inner diameter, there are limitations to the size of the particulate embolic agents and the type of coils that can be used through the 1.3-F microcatheter. Second, a triaxial system requires two microcatheters, which can complicate the procedure and increase costs.

## Conclusions

Transarterial embolization of an intercostal artery for T2EL after TEVAR is challenging. As seen in this case, a triaxial system with a 1.3-F small microcatheter is useful for T2EL embolization, particularly when the arterial route to the endoleak cavity is long and tortuous.
